# Advancement in the Antigenic Epitopes and Vaccine Adjuvants of African Swine Fever Virus

**DOI:** 10.3390/pathogens13080706

**Published:** 2024-08-21

**Authors:** Qiong Wu, Chang Li, Bo Zhu, Jiajia Zhu, Keli Yang, Zewen Liu, Wei Liu, Ting Gao, Fangyan Yuan, Rui Guo, Yongxiang Tian, Danna Zhou

**Affiliations:** Key Laboratory of Prevention and Control Agents for Animal Bacteriosis (Ministry of Agriculture and Rural Affairs), Hubei Provincial Key Laboratory of Animal Pathogenic Microbiology, Institute of Animal Husbandry and Veterinary, Hubei Academy of Agricultural Sciences, Wuhan 430064, China; wuqiong302@hbaas.com (Q.W.); lichang1113@hbaas.com (C.L.); 15972742735@163.com (B.Z.); xmszjj@hbaas.com (J.Z.); keliy6@hbaas.com (K.Y.); liuzwen2004@hbaas.com (Z.L.); liuwei@hbaas.com (W.L.); gaoting2017@hbaas.com (T.G.); fyyuan@hbaas.com (F.Y.); guorui@hbaas.com (R.G.)

**Keywords:** African swine fever virus, antigenic epitope, T-cell epitope, B-cell epitope, Vaccine adjuvants

## Abstract

African swine fever virus (ASFV), a highly virulent double-stranded DNA virus, poses a significant threat to global pig farming, with mortality rates in domestic pigs reaching up to 100%. Originating in Kenya in 1921, ASFV has since proliferated to Western Europe, Latin America, Eastern Europe, and most recently China in 2018, resulting in substantial global agricultural losses. Antigenic epitopes, recognized by the immune system’s T cells and B cells, are pivotal in antiviral immune responses. The identification and characterization of these antigenic epitopes can offer invaluable insights into the immune response against ASFV and aid in the development of innovative immunotherapeutic strategies. Vaccine adjuvants, substances that amplify the body’s specific immune response to antigens, also play a crucial role. This review provides an overview of the progress in studying T/B-cell epitopes in ASFV proteins and ASFV vaccine adjuvants, highlighting their role in the immune response and potential use in new vaccine development.

## 1. Introduction

African Swine Fever (ASF) is an acute hemorrhagic viral disease, caused by African Swine Fever Virus (ASFV), that affects domestic pigs and various species of wild boars [1,2]. ASFV is a member of the Asfarviridae family and is classified as a large icosahedral double-stranded DNA virus. Its linear genome spans approximately 189 kilobases and encodes for more than 180 genes. Since the 1960s, concerted efforts have been made to develop effective ASF vaccines, including ASF gene deletion vaccines, inactivated vaccines, multiepitope vaccines, attenuated live vaccines, and subunit vaccines [3,4,5]. Despite advancements, the absence of a clear understanding of immune correlates of protection and the precise identification of protective antigens has resulted in the current absence of an effective vaccine to halt the virus’s spread in pig farming. Consequently, it is essential to conduct comprehensive research into ASFV’s protective antigens, antigenic epitopes, and innovative vaccine adjuvants.

Much like most viral infections, both humoral immunity and cellular immune responses play crucial roles in ASFV protective immunity [6]. The serum from pigs in the recovery phase of ASFV infection possessed the ability to neutralize the infection of homologous and some heterologous strains both in vivo and in vitro, potentially by inhibiting virus attachment and internalization [7,8,9]. Anti-ASFV antibodies typically became detectable around six days post-infection, and could persist for an extended period once they had survived. However, despite the presence of these antibodies, their ability to cross-neutralize in vitro did not correlate with ASFV cross-protection in pigs [10]. Thus, the specific role of antibodies in ASFV protection remains unclear. ASFV serogroup classification, based on erythrocyte adsorption inhibition tests, suggests that ASF protective immunity may be serotype-specific, as ASFV within the same serogroup can cross-protect each other, while viruses outside the serotype cannot. Furthermore, a wealth of data supports the pivotal role of T-cell immune responses in ASFV control [6,11,12,13]. Depletion of pig lymphocytes indicated that cytotoxic CD8 lymphocytes were vital for ASFV clearance and protection against the virus. Moreover, pigs vaccinated with DNA vaccines exhibited partial protection during an ASFV attack, even though no anti-ASFV antibodies were detected in the protected pigs. Additionally, the failure of adjuvant-formulated inactivated ASFV and recombinant vaccines to offer strong protection highlighted the critical importance of cytotoxic T lymphocytes (CTLs) in the protective immune response against the virus [14].

Antigenic epitopes, the unique structural features on antigen molecules, exhibit specific antigenic functions. They are differentiated into B-cell and T-cell epitopes based on their interactions with corresponding antigen receptors on immune cells [15]. The investigation of antigenic epitopes is crucial for elucidating virus-induced immune responses and forms the foundation for the development of antiviral strategies, thereby representing a dynamic field of research in virology. According to the Immune Epitope Database (IEDB), several ASFV epitopes have been identified [16]. Despite the significant progress in ASFV antigenic epitope research, which has advanced the development of antigenic epitope diagnostic methods and vaccines, the identification and application of ASFV antigenic epitopes continue to pose challenges.

Vaccine adjuvants are substances that can enhance the body’s specific immune response to antigens [17,18,19]. When formulated with vaccines, they can effectively enhance the immune response to vaccine antigens. Adjuvants were first discovered in 1920 by French scientist Gaston Ramon. They usually do not have immunogenicity, but can guide humoral and cellular immune responses to produce specific immunity against pathogens [19]. Currently, adjuvants are used to enhance the immune response of vaccines and effectively reduce the dose of vaccine antigens, which is crucial in the production process of veterinary vaccines.

In this review, we mainly focus on the recent research progress of ASFV’s T-cell epitopes, B-cell epitopes, and vaccine adjuvants, aiming to provide basic information for the formulation of ASFV vaccine development and control strategies.

## 2. Advancement in the Development of Antigenic Epitopes of ASFV

### 2.1. Antigenic Epitope-Identified Proteins in ASFV

**Figure 1:**
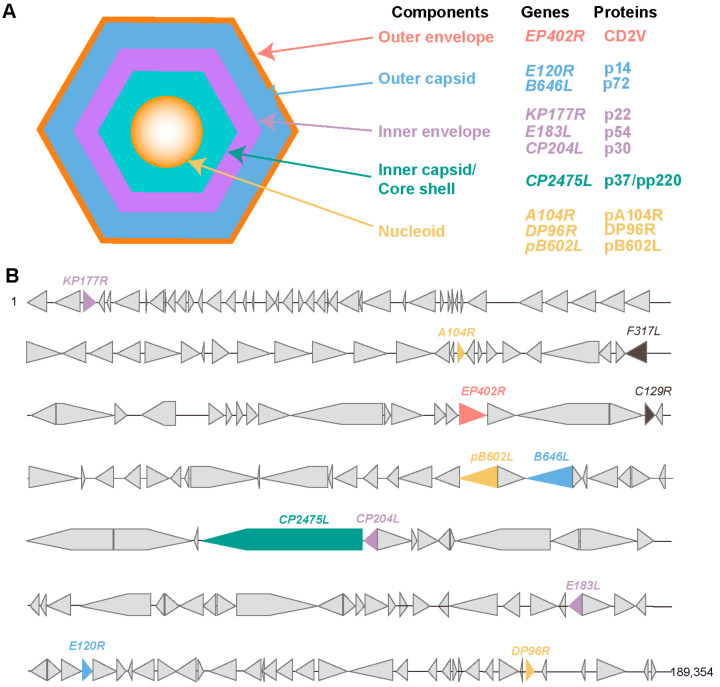
Schematic representation of ASFV structure and location of antigenic epitope-identified proteins in ASFV genomes. (**A**), Schematic diagram of the ASFV structure. ASFV is composed of an Outer envelope, outer capsid, inner envelope, inner capsid, and nucleoid. (**B**), Location of antigenic epitope-identified proteins in ASFV genomes.

The ASFV virion primarily consists of five components, including the viral genome, core shell, inner envelope, capsid, and outer envelope. The ASFV genome encodes approximately 150 to 200 proteins, of which about 60 are structural proteins. A large body of literature has confirmed the presence of B-cell and T-cell epitopes in ASFV. These antigenic epitope-identified proteins include p72, p37, p30, and CD2v (Figure 1).

### 2.2. Current Methods for the Identification of Antigenic Epitopes of the ASFV

#### 2.2.1. Methods for the Identification of B-Cell Epitopes

ASFV protein B-cell epitope identification is a systematic procedure involving protein expression, mouse immunization, monoclonal antibody production, and bioinformatics-guided epitope analysis. Epitope validation is further accomplished via peptide synthesis, alanine scanning in conjunction with Enzyme-Linked Immunosorbent Assay (ELISA), and dot-ELISA characterization. These methodologies have led to the discovery of numerous B-cell epitopes, including the extracellular domain of CD2v and p37 [20,21]. These findings are pivotal for elucidating immune response mechanisms against ASFV and facilitating effective vaccine development.

Given the multitude of ASFV proteins, predicting B-cell epitopes using bioinformatics tools remains an important and challenging task [22]. The complexity inherent to antigen recognition complicates epitope prediction. For linear B-cell epitopes, a prevalent approach involves the use of the ABCpred server, trained on the Bcipep database via a recurrent neural network (RNN), for analysis and selection. Epitopes with higher scores indicate a greater likelihood of B-cell epitope prediction. Subsequent secondary structure prediction is performed using DNAStar Protean software 11. The protein’s secondary structure significantly impacts the epitope. The robust chemical bonds of α-helices and β-sheets allow for the maintenance of protein structure, making such epitopes less likely to be antigenic due to their internal location and difficulty in antibody fitting. Conversely, the flexibility of β-turns and random coils in proteins allows for a looser structure, easily twisted and displayed on the protein surface. Such structures are more likely to serve as antigenic epitopes due to their prominence and ease of antibody fitting. For instance, Song et al. utilized ABCpred and DNAStar to predict the B-cell epitopes of pB602L, p30, p72, and CD2v proteins [12]. They evaluated the affinity of ASFV positive serum for synthetic peptides through peptide synthesis and dot blot analysis, successfully identifying immunodominant B-cell epitopes and incorporating them into nanoparticle vaccine antigen design [12].

#### 2.2.2. Methods for the Identification of T-Cell Epitopes

The most common identification process for T-cell epitopes in ASFV proteins includes ASFV protein expression, mouse immunization, IFN-γ ELISpot identification of cellular immune responses in splenic cells, bioinformatic analysis of T-cell epitopes, peptide synthesis combined with IFN-γ ELISpot, flow cytometry, and T-cell proliferation for further confirmation of T-cell epitopes. For instance, one T-cell epitope, ^246^ SRRSLVNPWT ^255^, has been identified on F317L using this method [23].

The use of bioinformatics tools for predicting ASFV T-cell epitopes is gaining traction. The most common approach for T-cell epitope prediction involves the use of software such as NetMHC 4.0 to screen potential T-cell epitopes. This software can predict peptides that bind with a multitude of SLA-I class molecules, from which high-affinity binding peptides are selected for biological synthesis and further epitope confirmation via IFN-γ ELISpot. For instance, Song et al. utilized NetMHC 4.0 to screen potential T-cell epitopes from CD2v and p72, and evaluated the cellular immune response of mice immunized with recombinant CD2v protein through peptide synthesis and IFN-γ ELISpot [12]. This validated the immunodominant T-cell epitopes, leading to the successful selection of immunodominant T-cell epitopes that were subsequently incorporated into the antigen design of nanoparticle vaccines.

### 2.3. Research Progress on B-Cell Epitopes in ASFV Proteins

#### 2.3.1. p72-Derived B-Cell Epitopes

Given the complexity of the p72 trimer structure, information about specific p72 epitopes remains limited. Previous studies have identified a linear B-cell epitope located at ^280^FPENSHNIQTAGKQD^294^ [24] and ^281^PENSHNIQTA^290^ [25]. Recently, Tesfagaber et al. prepared anti-p72 mAb and identified a new linear B-cell epitope region between ^283^NSHNIQ^288^ [26]. The identification of B-cell epitopes of p72 not only plays an important role in ASF serological diagnosis but may also lay a solid foundation for further research on the antigenic function of the p72 protein.

#### 2.3.2. CD2v-Derived B-Cell Epitopes

Multiple linear B-cell epitopes in the extracellular domain of ASFV’s CD2v have been identified through monoclonal antibodies (mAbs). For instance, a linear epitope formed by amino acids ^28^LDSNITNDNNINGVSWNFFNNSF^51^ was identified by mAbs produced by truncated CD2v protein fused with Norovirus (NoV) P particles [27]. Using mAbs produced by eukaryotic cells expressing CD2v, two epitopes, ^38^DINGVSWN^45^ and ^134^GTNTNIY^140^, were identified through peptide scanning [28]. Two linear B-cell epitopes, ^128^TCKKNNGTNT^137^ and ^148^VKYTNESILE^157^, were recognized by mAbs produced by truncated CD2v expressed in CHO-S [29]. The B-cell epitope (^154^SILE^157^) of the CD2v extracellular domain was further ensured by dot-Blot, ELISA and IFA tests [20]. Jia et al. identified three linear B-cell epitopes, ^147^FVKYT^151^, ^157^EYNWN^161^, and ^195^SSNY^198^, by screening five types of mAbs produced by truncated CD2v protein expressed in baculovirus [30]. Song et al. recently predicted and identified a major linear B-cell epitope, ^160^WNNSNINNFT^169^, which induced humoral and cellular immune responses in a mouse model, strongly suggesting that linear B-cell epitopes may promote the design and development of ASFV subunit vaccines [31]. Lu et al. identified a novel epitope, ^264^EPSPREP^270^, located in the CD2v-IR structural domain, which could be used for the design and development of subunit vaccines [32]. It is noteworthy that some of the linear B-cell epitopes mentioned above overlap; therefore, the immunogenicity of these epitopes needs to be evaluated.

#### 2.3.3. p30-Derived B-Cell Epitopes

p30 is a protein capable of generating neutralizing antibodies [33], with its C-terminus confirmed as an immunodominant region (aa 111–130) [34]. Petrovan et al. preliminarily identified a large polypeptide fragment (120–204 aa) at the C-terminus of the p30 protein by mAb 62-35 and 142-4 [35]. Recently, a study expressed overlapping truncated fragments in E. coli, determining that the linear epitope sequence recognized by monoclonal antibody 1B4G2–4 of p30 was within the range of ^157^FNKVIRAHNFIQTIYGTPLK^177^. After conducting amino acid-to-amino acid transfer identification, the smallest linear epitope was finally identified as ^164^HNFIQTI^170^. This epitope exhibited a strong antigen index and partial alpha-helical angles and coiled regions. It also showed high conservation across different strains, making it suitable for subsequent vaccine development [36].

#### 2.3.4. p54-Derived B-Cell Epitopes

Envelope protein p54, similar to B646L, can induce neutralizing antibodies in pigs, although these antibodies cannot provide protection against potent ASFV attacks [37]. Utilizing high-throughput analysis technology based on gene chips, Desmet et al. identified two B-cell epitopes of p54 (IVLIYLFSSRKKKAA and AA 149–161) [38]. Zheng et al. identified a novel linear B-cell epitope (^110^TMSAIENLR^118^) on the ASFV P54 protein using monoclonal antibodies, which was conserved in all reference ASFV strains from different regions in China [39]. Nanobodies were used as a new tool to identify linear B-cell epitopes on the ASFV p54 and a novel minimal linear B-cell epitope, ^76^QQWVEV^81^, was identified with core binding site as ^76^QQWV^79^ [40].

#### 2.3.5. DP96R-Derived B-Cell Epitopes

DP96R, also known as uridine kinase (UK), encodes a protein associated with virulence, which can be utilized in the development of attenuated live vaccines [41,42,43]. Two B-cell epitopes (^03^THDCSLKEK^11^ and ^55^YWKGIKRGND^64^) were found on ASFV’s DP96R protein [44].

#### 2.3.6. E120R-Derived B-Cell Epitopes

E120R is highly conserved among ASFV strains and serves as a target for the development of attenuated live vaccines against ASF [45]. Through incubation with a gene chip based on high-density peptide microarrays, it was discovered that the peptide sequence EEFEPIPDYDTTST of ASFV envelope protein E120R could react with ASF positive serum samples [38].

#### 2.3.7. pA104R-Derived B-Cell Epitopes

The A104R gene is responsible for the synthesis of a protein, hypothesized to resemble a histone, which is strategically positioned at the sites of viral DNA replication and gene expression [46]. The dominant IgM epitope PEP23 (KAVKIRALK) and PEP15 (KFTVVTVKA) were identified by epitope modification [47]. Through the confirmatory analysis of the pA104R epitope using monoclonal antibodies, an immunodominant B-cell epitope (KPTITKOELYSI) was identified. This finding could potentially aid in the development of sensitive diagnostic tools and serve as a target for candidate vaccine development [48].

#### 2.3.8. E184L-Derived B-Cell Epitopes

E184L serves as a crucial antagonist of the IFN signal and an immunogenic ASFV protein, capable of evading the host’s innate antiviral immune response [49,50]. Through meticulous localization, the antigenic epitopes for the E184L mAbs have been identified as ^119^ IQRQGFL ^125^ and ^153^ DPTEFF ^158^. These findings lay the groundwork for serological diagnosis and the development of epitope-based marker vaccines [51].

Taken together, numerous B-cell epitopes have been identified in ASFV, which guide the design of immunogenic peptides and novel vaccine molecules, and also facilitate the development of diagnostic reagents and clinical disease diagnosis (Table 1).

### 2.4. Research Progress on ASFV-Specific T-Cell Epitopes

#### 2.4.1. CD2v-Derived T-Cell Epitopes

The intracellular epitope in the CD2v protein of ASFV has been demonstrated to induce both humoral and cellular immune responses [32,56]. The CD2v protein’s proline-rich cytoplasmic domain demonstrated a high degree of conservation, with 79% to 100% amino acid identity, across various genotypes of ASFV [56]. The discovery of monoclonal antibody 1F3 (^264^EPSPREP^270^) has identified it as a T-cell epitope that was not only specific to ASFV but also remarkably conserved across different genotypes [32].

#### 2.4.2. p72-Derived T-Cell Epitopes

The p72 protein is the major capsid protein and also one of the most immunogenic proteins of ASFV, making it an important target for detection and vaccine development [57]. Using the IEDB MHC-I binding prediction algorithm coupled with ELISPOT assay detection, Sun et al. have delineated the core peptides P351 (SRISNIKNNKY), P334 (SDYTL), and P366 (SSYGGAK) derived from the p72 protein as exhibiting elevated immunogenicity in pigs that have survived infection, offering a pivotal reference for the subsequent development of tetrameric constructs in immunological research [58].

#### 2.4.3. F317L-Derived T-Cell Epitopes

The late F317L protein of ASFV induced immunosuppression by dampening the activation of the NF-ĸB pathway, making it a potential immunogenic antigen [59,60]. Through T-cell epitopes prediction and validation by the IFN-γ ELISpot assay, the peptide F25 (^246^SRRSLVNPWT^255^) was identified as a presumed T-cell epitope, capable of inducing a robust immune response [23].

#### 2.4.4. C129R-Derived T-Cell Epitopes

The C129R protein of ASFV, known for its strong immunogenicity and ability to target the cyclic GMP-AMP pathway, was utilized in the development of recombinant adenovirus vaccines [10,61]. Recently, Zhai and colleagues successfully pinpointed T-cell epitopes within the C129R protein, specifically in peptides C11 (^53^LQNPYEAVI^61^), C14 (^81^GHVTWAVPY^89^), C16 (^97^AKPDAIMLT^105^), and C18 (^116^ALNQNVLTL^124^) [62].

Collectively, the validated ASFV-specific T-cell epitopes summarized in this review may play a crucial role in the design and development of novel ASFV vaccines (Table 2).

## 3. Advancement in the Development of Vaccine Adjuvants of ASFV

### 3.1. The Functions and Mechanism of Adjuvants in ASF Vaccines

In the development of ASFV vaccines, the selection and use of adjuvants is a critical component. Adjuvants can enhance the immunogenicity of vaccines, improve the durability of immunity, reduce vaccine dosage, and thus improve the cost-effectiveness of vaccines [17,18,19]. Currently, a variety of adjuvants have been used in the development of ASFV vaccines, including aluminum salts, oil emulsions, and polymer microspheres [63,64]. These adjuvants were shown to enhance the immune response through various mechanisms, such as stimulating immune cells, promoting antigen uptake by antigen-presenting cells (APCs), stimulating the secretion of various cytokines and chemokines, inducing the differentiation of CD4 + T cells into different types (Th1, Th2, Th17, etc.), prolonging antigen presentation time, and enhancing antigen presentation efficiency [63,64].

### 3.2. Types of Adjuvants for ASF Vaccines

#### 3.2.1. Montanide™ ISA

Montanide™ ISA, the veterinary vaccine adjuvant, is a common choice in animal vaccines due to its ability to induce strong immune responses with non-toxicity, good tolerability, and the simplicity of mixing the antigen and adjuvant [65]. Zajac et al. used a recombinant adenovirus mixture containing ASFV antigens (Ad5-ASFV) with 42 multi-epitopes, combined with Montanide ISA-201™ adjuvant, to immunize pigs three times. This could induce a humoral immune response, but there was no protective effect after the immune challenge [64]. A recent study utilized an inactivated vaccine produced by gamma-irradiated ASFV in combination with Montanide™ ISA 201 VG adjuvant. This formulation was administered to five weaned piglets at three-week intervals for two immunizations. Despite all animals developing antibodies against ASFV p72, the response was insufficient to confer protection from ASFV strain attacks [66].

#### 3.2.2. Polygen™

Polygen™ is a low molecular weight copolymer adjuvant that can cross-link in solution to form a high molecular weight gel. It has been demonstrated in studies to elicit significant interferon-gamma (IFN-γ) and interleukin-12 (IL-12) responses when used in bovine parasitic vaccines [67]. Researchers administered an inactivated vaccine, prepared by combining ASFV inactivated by gamma irradiation or binary ethylenimine (BEI) with the Polygen adjuvant, via intramuscular injection to weaned piglets. Despite the presence of ASFV-specific antibodies in all vaccinated subjects, no protective effect was observed, even after stringent homologous challenge [66,68].

#### 3.2.3. Zoetis

Vaccine adjuvants produced by Zoetis have been used in vaccine immunization research for various pathogens, including *paratyphoid salmonella* and porcine reproductive and respiratory syndrome virus [69,70,71]. Pigs immunized with a cocktail of 12 ASFV antigens combined with the Zoetis adjuvant showed that the cocktail-ii- Zoetis vaccine recipients had a higher survival rate, but it did not prevent clinical disease [63]. Compared to the ENABL adjuvant, pigs vaccinated with Ad5-ASFV 4-way cocktail vaccine combined with Zoetis adjuvant exhibited higher humoral immune response [72].

#### 3.2.4. BioMize^®^

BioMize^®^ is an innovative, ready-to-use, and fully customizable adjuvant developed by VaxLiant company, offering great flexibility for vaccine development and commercialization [63,64,73]. Compared to the use of the Montanide ISA-201™ adjuvant, the Ad5-ASFV BioMize^®^ immunogen elicited a relatively lower anti-pp62 specific IgG response [64]. Immunizing pigs with a 35 adenovirus-vectored ASFV cocktail, along with BioMize^®^ adjuvant, generated robust ASFV-specific antibodies, IFN-γ cells, and CTL responses, yet failed to confer protection against the virulent Arm07 isolate in Eurasian wild boar [73].

#### 3.2.5. MF59^®^

MF59^®^ is an oil-in-water emulsion adjuvant that has been included in influenza vaccines approved in Europe since 1997 and has been administered to over 100 million people in more than 30 countries [74]. The BEI-inactivated ASFV vaccine, combined with the MF59 adjuvant and administered intradermally and intramuscularly in pigs, elicited a positive antibody response to ASFV. However, it did not provide effective protection against a lethal attack [75].

#### 3.2.6. Adjuvants from Bacterial Component

The major outer membrane lipoprotein I (OprI) of *Pseudomonas aeruginosa* is a ligand for Toll-like receptor (TLR)-2 [76]. It can trigger dendritic cells (DC) to secrete pro-inflammatory cytokines in vivo, thereby indirectly regulating adaptive immune responses. OprI can serve as a natural adjuvant, and after fusion, it can induce a strong humoral and cytotoxic T-cell response against peptides/proteins. The application of OprI in fusion proteins has been extended to antigens encoded by ASFV’s B646L and G1340L, and the resulting proteins can induce ASFV-specific CTL activity [76,77]. The different immune functions of OprI, including promoting Th1/Th2 responses, are all attributed to the activation of TLR-2 signaling. The immunoregulatory activity of OprI fusion proteins has also been used in vaccine development. A recent study has shown that the mixture of OprI fusion proteins formulated with ISA206 adjuvant can induce strong ASFV-specific humoral and cellular immune responses in pigs, providing valuable information for the further development of subunit vaccines against ASF [11]. Heat-labile enterotoxin B (LTB), when used as an adjuvant carrying antigens, can enhance the immunogenicity of vaccines and play a role in T-cell activation and differentiation [78]. Following oral immunization with recombinant Lactobacillus lactis expressing ASFV protein-LTB fusion protein, the local mucosal immunity, humoral immunity, and Th2 cell immunity were enhanced compared with no LTB adjuvant group [79]. These findings provide new insights into the design and development of ASFV subunit vaccines.

#### 3.2.7. Nano-Adjuvants

Compared to traditional vaccines, nano-vaccines demonstrate enhanced efficacy due to their ability to accumulate, self-assemble in lymph nodes, and be readily uptaken by APC cells [80]. Recently, a self-assembling nano ASFV candidate vaccine (NanoFVax) targeting dendritic cells has been demonstrated to elicit a potent T-cell response, with high-level antibody responses against ASFV persisting for over 231 days [12].

In summary, the selection and optimization of adjuvants for ASFV vaccines remains an important area of research that requires further exploration and study (Table 3).

## 4. Prospects of Antigenic Epitopes and Adjuvants in the Development of Novel ASFV Vaccines and Diagnostic Reagents

Currently, techniques for identifying B-cell epitopes are well established. However, obtaining high-quality monoclonal antibodies is challenging, especially those with neutralizing activity that can recognize conformational epitopes. For the recognition of conformational B-cell antigenic epitopes, monoclonal antibodies recognizing the whole virus are more effective than those recognizing recombinant proteins. Novel ASFV detection methods, such as ELISA and flow cytometry, rely on the identification of these B-cell epitopes. By recognizing and utilizing these epitopes, we can develop more sensitive and specific detection methods to facilitate early detection and diagnosis of ASFV infection, thereby effectively controlling the spread of ASF. The OIE recommended ELISA as the preferred serological method for ASF diagnosis [83]. Despite its lower sensitivity compared to highly sensitive early pathogen detection methods such as PCR, qPCR, multiplex PCR, LAMP, and NGS, the ELISA demonstrated significant advantages in large-scale sample testing. It was particularly suitable for detecting pig herds recovering from subacute and latent ASF infections [84]. Most commercialized ELISA kits were based on a single viral protein, which could lead to false-negative results. However, ASFV tandem proteins based on multiple B-cell epitopes showed significant improvements in sensitivity in the development of ELISA kits [85,86]. It enhanced the recognition of different types of antibodies in pigs, reduced the possibility of inaccurate negative or positive results, and allowed a comprehensive assessment of ASFV exposure by k3 derived from 27 multiple peptides of 11 ASFV proteins or the antigenic dominant domains from p30, p54, and p72 [85,86]. Compared with currently available commercial detection methods, innovative methods using multiple B-cell epitopes of multiple ASFV proteins can achieve higher sensitivity and specificity.

The identification of T-cell epitopes is crucial for research on cellular immune mechanisms and the development of subunit peptide vaccines. Apart from p30, p54, and p72, there are few reports on the research of T-cell antigenic epitopes of other proteins [23]. Since T-cells only recognize antigenic peptides presented by the Major Histocompatibility Complex (MHC) molecules on the surface of Antigen Presenting Cells (APCs), the recognition of T-cell epitopes is more challenging [87]. The development of ASFV vaccines that focused solely on humoral immune responses was insufficient, as inactivated vaccines had been shown to be ineffective in providing protection against ASFV challenge [68]. Attenuated or low-virulence ASFV strains induced protective immunity in pigs against virulent ASFV strains [88], but vaccinated pigs often experienced adverse side effects, such as chronic viremia [3]. Additionally, the use of live attenuated ASFV raised significant safety concerns [3]. Furthermore, the activation of CD8 T cells aided pigs in combating ASFV infection [89], underscoring the crucial role of antigen-specific T cell immune responses in ASFV vaccine development. This necessitates further investigation into ASFV’s T-cell epitopes.

Although inactivated and subunit vaccines for ASFV have a high safety profile and have been used in conjunction with some adjuvants, they have not demonstrated robust protective effects in current research [3,90,91]. This indicates that there are many unknowns that need to be addressed with protective antigens or epitopes, adjuvants, and non-structural viral protein antigens in the development of new ASFV vaccines. Although the only attenuated live vaccine approved by Vietnam demonstrated that 93.34% of the 5958 randomly selected immunized pigs met the technical requirements, as reported in the June 2023 Global Disease Monitoring Report from the Swine Health Information Center, potential biosecurity risks such as reversion to virulence necessitated caution [91]. Therefore, the development of new nucleic acid vaccines, genetically engineered vaccines, or multi-peptide vaccines in combination with new adjuvants is an important direction for current ASFV vaccine development. When choosing a vaccine adjuvant, many factors need to be considered, with safety being the first. A good adjuvant must be safe, well-tolerated, and easy to produce; it should have good pharmaceutical properties (such as pH, osmotic pressure, endotoxin levels, etc.) and long shelf life; and finally, it should be economically viable.

Reverse vaccinology is an application that aids in the development of novel epitope vaccines based on pathogen genome sequencing [92]. Compared to traditional vaccines, epitope vaccines are safer, non-toxic, stable, and can more directly elicit immune responses against pathogenic microorganisms [93]. Epitope vaccines based on multiple epitope peptides can also overcome the problem of low conservation between epitopes of different genotype strains and elicit stronger immune responses. However, for ASFV, the development of diagnostics and vaccines based on multiple epitopes is still insufficient [94,95]. As multi-epitope vaccines are based on the selection of antigenic epitopes and the immune response of computer-screened epitopes, they are suitable for the development of universal vaccines against different ASFV genotypes, accelerating the vaccine design process and reducing its cost.

## 5. Conclusions

African swine fever infection causes high mortality in pigs, resulting in significant economic losses to the pig industry. Research on African swine fever vaccines has been ongoing in recent decades. The antigenic epitopes recognized by T cells and B cells of the immune system play a key role in the antiviral immune response, and therefore the resolution and precise characterization of the T-cell antigenic epitopes and B-cell antigenic epitopes of ASFV can provide an important basis for vaccine development. Here, we describe the major potential antigenic proteins of ASFV and methods for pinpointing T- and B-cell epitopes of ASFV antigens, providing detailed localization data for these epitopes. These will hold promise for the development of safe and effective ASF vaccines. Vaccines, combined with accurate, efficient, and early diagnostic techniques, could provide the basis for the prevention, control, and eradication of ASF.

## Figures and Tables

**Table 1:** Identification and application of B-cell epitopes of ASFV antigens.

ASFV (Protein)	Sequence	Application		References
		Vaccine	Diagnosis	
p72	^156^TLVDPFGRPI^165^ ^265^QRTCSHTNPKFLSQHF^280^ ^280^FPENSHNIQTAGKQD^294^ ^290^AGKQDITPITDATY^303^	−	+	[24]
	^281^PENSHNIQTA^290^	−	+	[25]
	^283^NSHNIQ^288^	−	+	[26]
CD2v	^154^SILE^157^	+	+	[20]
	^28^LDSNITNDNNINGVSWNFFNNSF^51^	−	+	[27]
	^38^DINGVSWN^45^ ^134^GTNTNIY^140^	+	−	[28]
	^128^TCKKNNGTNT^137^ ^148^VKYTNESILE^157^	−	+	[29]
	^147^FVKYT^151^ ^157^EYNWN^161^ ^195^SSNY^198^	+	+	[30]
	^160^WNNSNINNFT^169^	+		[31]
	^264^EPSPREP^270^	+	+	[32]
	^157^FNKVIRAHNFIQTIYGTPLK^177^	+	−	[36]
p54	^149^IVLIYLFSSRKKKAA^161^	+	−	[38]
	^110^TMSAIENLR^118^	+	−	[39]
	^76^QQWVEV^81^	+	−	[40]
p37	^58^LGDAIAGRLMQLD^70^ ^100^DWKATVSAIELEY^112^ ^163^TTGDTLAQVFESFPT^177^ ^63^AGRLMQLDTEKNARI^77^	+	−	[21]
E120R	EEFEPIPDYDTTST	+	−	[38]
DP96R	^03^THDCSLKEK^11^ ^55^YWKGIKRGND^64^	−	+	[44]
pA104R	KPTITKOELYSI	+	+	[48]
	KFTVVTVKA KAVKIRALK	+	−	[52]
E184L	^119^IQRQGFL^125^ ^153^DPTEFF^158^	+	+	[51]
pB602L	^474^SKENLTPDE^482^	+	+	[12,53]
p22	^37^KVCKVDKDCGSGEHC^51^ ^157^VYNNPHHPVLKYGKDHIIALP^171^	−	+	[54]
MGF_110-13L	^48^WDCQDGICKNKITESRFIDS^67^ ^122^GDHQQLSIKQ^131^	−	+	[55]

**Table 2:** Identification and application of T-cell epitopes of ASFV antigens.

ASFV (Protein)	Sequence	Application		Reference
		Vaccine	Diagnosis	
F317L	^246^SRRSLVNPWT^255^	+	−	[23]
CD2v Intracellular Epitope	^264^EPSPREP^270^	+	−	[32]
p72	P351(SRISNIKNNKY) P334(SDYTL) P366(SSYGGAK)	+	−	[58]
C129R	^53^LQNPYEAVI^61^ ^81^GHVTWAVPY^89^ ^97^AKPDAIMLT^105^ ^116^ALNQNVLTL^124^	+	−	[62]

**Table 3:** Research progress on adjuvants in ASFV vaccines.

Adjuvant Name	The Components of ASFV Vaccine	Immune Rout	Reference
Montanide ISA	OPMT + OPET + OCET + MontanideTM ISA206™	i.m.	[11]
	Ad5-ASFV Mix + Montanide ISA-201™	i.m.	[64]
	30 kGy Irradiated ASFV + Montanide ISA-201™	i.m.	[66]
	Inactivated ASFV + MontanideTM ISA201™	i.m.	[75]
Polygen™	30 kGy Irradiated ASFV + Polygen™	i.m.	[66]
	BEI-inactivated ASFV + Polygen™	i.m.	[68]
Zoetis	Ad-ASFV cocktail-II + Zoetis	i.m.	[63]
	Ad5-ASFV 4-way cocktail + Zoetis	i.m.	[72]
ENABL	Ad5-ASFV 4-way cocktail + ENABL	i.m.	[72]
BioMize^®^	Ad-ASFV cocktail-I + BioMize	i.m.	[63]
	Ad5-ASFV + BioMize	i.m.	[73]
MF59^®^	Inactivated ASFV + MF59^®^	i.m.	[75]
Emulsigen D	BEI-inactivated ASFV + Emulsigen D	i.m.	[68]
Silica oil	Inactivated ASFV + Silica oil	i.m.	[75]
mGNE	Inactivated ASFV + mGNE	i.m.	[75]
LTB	MG1363/pMG36e-p30 + p54 + p72-LTB-His	Oral	[79]
OprI	OPMT + OPET + OCET + OprI	i.m.	[11]
FlaB	rAd-ASFV CD2v-p30-p54-FlaB	i.n.	[81]
Hsp70	rAd-ASFV CD2v-p30-p54-Hsp70	i.n.	[81]
IL-33	NC8-pLP-S-p14.5-IL-33-Mus	Oral	[82]
CTA1-DD	NC8-pLP-S-CTA1-p14.5-CTA1-DD	Oral	[82]
SpyTag-SpyCatcher	SpyTag/SpyCatcher + ASFV epitopes + SpyTag-SpyCatcher	i.m.	[12]

i.m.: intramuscular immunization; i.n.: intranasal immunization.

## Data Availability

No new data were created or analyzed in this study. Data sharing is not applicable to this article.
